# Health Related Quality of Life of Ugandan Children Following Valve Replacement Surgery for Rheumatic Heart Disease

**DOI:** 10.5334/gh.1205

**Published:** 2023-06-23

**Authors:** Mohammed A. M. Ahmed, Twalib Aliku, Judith Namuyonga, Bernard Obongonyinge, Hilda Tumwebaze, Samalie M. Kitooleko, Tom Mwambu, Peter Lwabi, Sulaiman Lubega

**Affiliations:** 1 Department of Paediatric Cardiology, Uganda Heart Institute, Kampala, Uganda; 2 Department of Paediatrics, Faculty of Medicine and Surgery, Mogadishu University, Mogadishu, Somalia; 3 School of Medicine, Uganda Christian University, Mukono, Uganda; 4 Department of Adult Cardiovascular Surgery, Uganda heart institute, Kampala, Uganda

**Keywords:** Rheumatic heart disease, valve replacement surgery, health related quality of life, Uganda

## Abstract

**Background:**

Valve replacement surgery (VRS) improves clinical outcomes in patients with severe rheumatic heart disease (RHD). However, lifelong anticoagulation and frequent monitoring are required, which potentially impacts health-related quality of life (HRQoL). In this study, we assessed the HRQoL of people with RHD in Uganda following VRS.

**Methods:**

This was a hospital-based, cross-sectional study conducted between March and August 2021. Eligible participants were individuals who had VRS before the age of 18 years. The Pediatric Quality of Life Inventory–Cardiac Module (PedsQL-Cardiac module) was used to evaluate HRQoL. A total mean score of ≥ 80% was considered as optimal HRQoL.

**Results:**

Of the 83 eligible participants, 52 (60.5%) were female, with a median age of 18 (interquartile range: 14–22) years. Most participants had NYHA I functional status (n = 79, 92%). Most (n = 73, 92.4%) surgeries were performed outside of Uganda, and 61 (72.6%) were single mechanical valve replacement. Almost half (n = 45, 54%) expressed no concern about being on life-long warfarin therapy. However, 24 (29.3%) feared bleeding. The optimal mean score of cardiac-specific HRQoL was achieved in 50 (60.2%) of participants. Factors associated with optimal HRQoL were body mass index (BMI) (adjusted odds ratio (aOR), 1.2, 95% Confidence Interval: 1.1–1.3, p = 0.006), being afraid of bleeding or bruising (aOR: 1.5, 95% CI: 1.21–2.47, p = 0.004), acceptance of having an artificial valve (aOR: 2.7, 95% CI; 1.64–3.81, p < 0.001).

**Conclusion:**

HRQoL was optimal in about three in five participants following VRS. Increasing BMI and acceptance of artificial valve were significantly associated with optimal HRQoL.

## Introduction

Rheumatic heart disease (RHD) is the most common preventable and treatable form of cardiovascular disease in children and young adults [1]. RHD is highly endemic in poor, most vulnerable populations and imposes heavy costs on the health systems particularly in low- and middle-income countries [1]. At the end of 2017, approximately 40 million people worldwide were estimated to be living with RHD, leading to the death of about 300 000 cases each year [2].

Valve replacement surgery (VRS) in RHD is associated with improvement in functional status [3]. Two types of valves are commonly used: namely, bio-prosthetic and mechanical (or metallic) [4]. Mechanical valves are usually preferred in resource-limited settings over bio-prosthetic valves, mainly because bio-prosthetic materials are less durable and often require replacement in about 10 years which may not be affordable. Despite being durable, the mechanical valves are highly thrombogenic and require lifelong anticoagulation (typically with warfarin) with frequent monitoring of the international normalized ratio (INR) to ensure therapeutic anticoagulation [4].

The Uganda Heart Institute (UHI) has comprehensive primary and secondary RHD prevention programs and an established national RHD register for children and adults with RHD [5]. However, the number of children who access VRS is significantly limited by low funding and the absence of specialized sundries like valve implants. Local cardiac surgeons, and occasionally visiting experts perform VRS at UHI, but most children have their VRS done from outside of the country funded by non-governmental organizations or individual families.

VRS generally improves life expectancy, quality of life and functional status [3]. The success rates following VRS are generally measured based on post-operative mortality, morbidity and outcomes of the patients [3,6]. However, the impact of VRS on health-related quality of life (HRQoL) is equally important [7].

Long-term warfarin prophylaxis with regular INR monitoring is standard of care following VRS and may impact HRQoL. The American Heart Association (AHA) considers HRQoL evaluation as a strategic treatment impact goal for cardiovascular health [8] and subsequently defines HRQoL as the “discrepancy between actual and desired functional status and overall impact of health on well-being” [9].

A study in the United Kingdom reported non-perception of normal health by the parents and children following mitral valve replacement, and the level of HRQoL impairment was equal to or worse than that previously published for children with chronic disabilities [10]. Moreover, evaluation of HRQoL in children with VRS can identify hidden morbidities or challenges that might be addressed by the healthcare provider and assist in clinical decision-making [11]. However, there is a scarcity of literature on the impact of VRS and the long-term anticoagulation on the HRQoL following VRS in resource limited settings like sub-Saharan Africa. Therefore, we aimed to assess the HRQoL of children with (RHD) in Uganda following VRS.

## Methods and Materials

### Study site

The study was conducted at the UHI, a public, tertiary level cardiovascular referral facility in Kampala, Uganda, and one of the training centers for the College of Health Sciences, Makerere University.

### Study design

This was a cross-sectional study conducted between April and August 2021 at the UHI, Pediatric Cardiology Clinic.

### Selection criteria

We recruited children above eight years of age and adults who had VRS before the age of 18 years, after the first six months of surgery. We excluded participants with life-threatening cardiac emergencies requiring urgent interventions or hospitalization.

### Sample size

**Figure 1:**
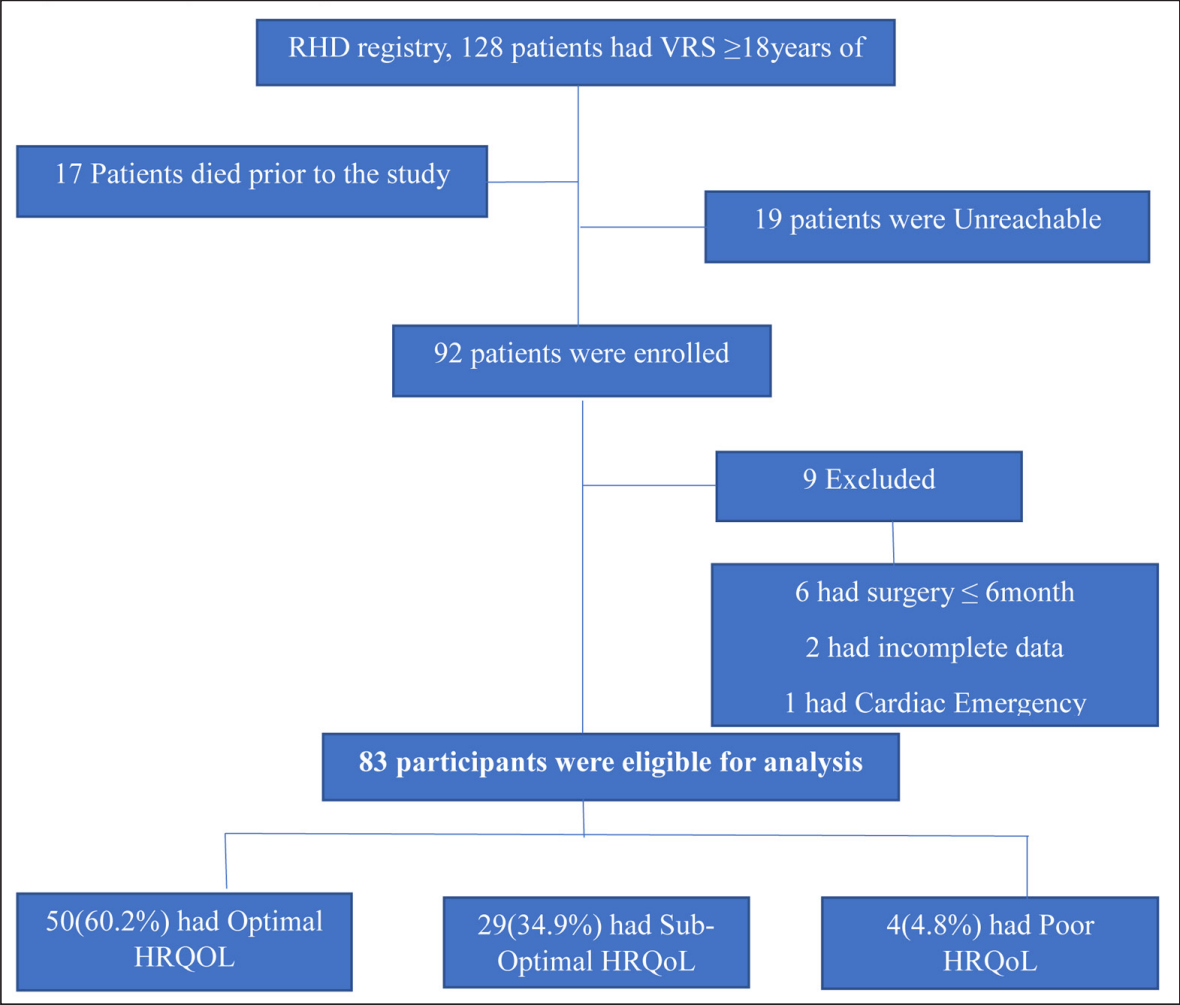
Study Flow Diagram.

We assessed for eligibility and considered all participants in the UHI RHD registry, including follow-up phone calls to all potential participants whose appointment dates were not within the study period (Figure 1).

### Study Procedures

All participants in the RHD registry that met inclusion criteria were contacted by telephone to come for participation into the study and were enrolled in the study. The study was explained to the subjects and written informed consent and assent were obtained from the study participants. The survey was conducted using an interviewer-administered questionnaire. The research assistant (either a nurse or physician) verbally asked questions to the participants and then recorded their responses. Participants aged less than 18 years were interviewed together with the parent or caretaker. Participants aged over 18 were interviewed separately. The coronavirus disease-2019 (COVID-19) standard operating procedures set by the Ministry of Health, Uganda, were strictly adhered to throughout the study. Study staff was equipped with personal protective equipment such as a facemask and a hand sanitizer.

### Study tool: PedsQL 3.0 cardiac module

We used the PedsQL 3.0 cardiac module that has 22 items under five scales—symptoms, perceived physical appearance, treatment anxiety, cognitive problems and communication [12] In addition, a treatment barriers scale is included for patients on medications. A 5-point Likert response scale (0–4) is used for scoring responses from the participants. All domains were reverse-scored and transformed to a 0–100 scale (0 = 100, 1 = 75, 2 = 50, 3 = 25, 4 = 0) for easy interpretation. Component scale scores were then calculated as the sum of the items divided by the number of items answered. The higher the scores on the scale signify the better HRQoL, we used the cut off ≥80% for optimal HRQoL, ≥70% – ≤80% for sub optimal and ≤70% for poor HRQoL as previously described [13]. To examine the effect of regular blood testing and continuous use of blood thinners further questions were added from the literature [10]. We used the New York Heart Association (NYHA) for functional status classification [14], and international reference tables for body mass index (BMI) interpretation [15].

### Statistical Analysis

The data was collected using paper forms, then entered Microsoft Excel (2016) for cleaning and coding then exported to STATA version 16 (StataCorp LLC., College Station, Texas, USA) for analysis. Continuous variables were presented either as means and standard deviation or median and interquartile ranges (IQR). For normally distributed data, independent sample (student) t-test was used to compare for differences, meanwhile, non-normally distributed data were compared using Mann-Whitney U test. Categorical data was presented as frequencies and percentages and compared using Fischer’s exact test or Chi-square test as appropriate all variables with a p < 0.2 at bivariate analysis were forwarded in the final logistic regression analysis model to determine independent predictors of HRQoL. Results were presented as adjusted odds ratio (aOR), with corresponding 95% Confidence Interval (CI). A p < 0.05 was considered statistically significant.

## Results

### Socio-demographic characteristics of the study participants

In total, data of 83 eligible patients was analysed. Of this, 49 (59%) were female and the median age at surgery was 14 (IQR: 7–18) years. Most (71, 89%) of the VRS were performed in Sudan followed by Uganda (6, 7.5%) and India (2, 2.5%). Nearly three quarters (58, 72%) of participants had a single mechanical valve. The median duration of RHD from first diagnosis to the time of surgery was 6 (IQR: 2–25) years. The median time to have VRS was 2 (IQR: 1–4.8) years (Table 1). Almost all the participants (n = 79, 92%) reported no limitation to ordinary activities (NYHA I), while 3 (8%) reported slight limitations (NYHA II).

**Table 1:** Socio-demographic characteristics of the participants.


VARIABLE (N = 83)	*N(%)*

Age, mean (SD), years	18.5(4.5)

<19	42(50.6)

> = 19	41(49.4)

Weight, mean (SD), kg	51.9(11.3)

Height, mean (SD), cm	158.6(8.6)

Gender	

Female	49(59)

Male	34(41)

Level of education	

Pre–primary	4(4.8)

Primary	36(43.4)

Secondary	39(47)

None	4(4.8)

SPO2, median (IQR), percentage	99(96–100)

Heart rate, mean (SD), BPM	84.5(10.8)

Duration of heart disease from 1^st^ diagnosis, median (IQR), years	6(2–25)

Duration from surgery, median (IQR), years	4(2–6)

Time to surgery from initial diagnosis, median (IQR) years	2(1–4.8)

Underweight (below 18.5)	26(31.3)

Normal weight (18.5–24.9)	46(55.4)

Overweight (25.0–29.9)	9(10.8)

Obese (30.0 and above)	2(2.4)

Country of surgery	

India	2(2.5)

Uganda	6(7.5)

Sudan	71(88.8)

Type of valve replacement surgery	

Single valve	58(71.6)

Double valve	23(28.4)

Age at surgery (years)	14(7–18)


### Feelings of the study participants about warfarin use and regular blood testing (PT/INR)

Just above half (n = 45, 54%) of the participants reported no problems with taking warfarin and most (n = 70, 85.5%) reported a full understanding of the use of warfarin. Overall, 38 (45.8%) participants reported difficulties in regular blood testing and 41 (50%) could not afford it (Table 2).

**Table 2:** The feelings of the participant about warfarin use and regular blood testing (PT/INR).


VARIABLE (N = 83)	DEFINITELY TRUE	MOSTLY TRUE	NOT SURE	MOSTLY FALSE	DEFINITELY FALSE

Taking warfarin					

I don’t mind taking warfarin	45(54.2)	0(0)	0(0)	0(0)	38(45.8)

I can’t always be bothered taking my tabs	21(25.3)	0(0)	0(0)	0(0)	62(74.7)

It interferes with my social life	15(18.1)	1(1.2)	0(0)	1(1.2)	66(79.5)

I don’t fully understand why I have to take them	4(5)	0(0)	3(3.8)	3(.3.8)	70(85.5)

I simply forget	7(8.5)	1(1.2)	2(2.4)	3(3.7)	69(84.2)

I am afraid I may get a bleed or bruise	24(29.3)	1(1.2)	1(1.2)	3(3.7)	53(64.6)

I don’t want my friends/colleagues to know I have an artificial valve	35(42.2)	1(1.2)	0(0)	0(0)	47(56.6)

**DOING REGULAR BLOOD TESTS**					

I don’t mind having regular blood tests	44(53)	0(0)	0(0)	1(1.2)	38(45.8)

The tests are painful	40(48.2)	0(0)	2(2.4)	2(2.4)	39(47)

It interferes with my daily life	17(20.5)	0(0)	2(2.4)	1(1.2)	63(75.9)

I don’t understand why I have to have them	8(9.6)	0(0)	2(2.4)	1(1.2)	72(86.8)

It is expensive for me (I can’t afford it)	41(50)	1(1.2)	2(2.4)	0(0)	38(46.3)


### Healt-Hrelated quality of life of the participants

The mean cardiac-specific HRQoL score was 83.4 ± 12.9%. Overall, 50 (60.2%) participants had optimal self-reported HRQoL. The participants best scored in treatment barriers 91.1 ± 14.8%, treatment anxiety 90.4 ± 20.5%, cognitive problems at 90.6 ± 15.1%, and the worst score was for the perceived physical appearance at 55.8 ± 41.8% (Table 3).

**Table 3:** Health-related quality of life for all participants in the study.


DIMENSION	MEAN (SD)

Heart Problems and Treatment	85.1 (14.2)

Treatment Barriers	91.1 (14.8)

Perceived Physical Appearance	55.8 (41.8)

Treatment Anxiety	90.4 (20.5)

Cognitive Problems	90.6 (15.1)

Communication	87.5 (22.4)

Health-Related Quality of Life, Total Score	83.4 (12.9)

**Health-Related Quality of Life, classification**	**Freq (%)**

Optimal	50(60.2)

Sub-optimal	29(34.9)

Poor	4(4.8)


Optimal mean HRQoL score ≥ 80%, Sub optimal ≥ 70% – ≤80% and poor HRQoL ≤ 70%.

### Factors associated with optimal healt-Hrelated quality of life

At bivariate analysis, patients fear for getting bleeding or bruising from warfarin side effects, willingness to tell others about the presence of an artificial valve and BMI were statistically significantly associated with optimal HRQoL (Tables 4a and 4b).

**Table 4a:** Socio-demographic factors associated with optimal health-related quality of life of the participants at bivariate analysis.


VARIABLE	ALL (N = 83) FREQ (%)	HEALTH RELATED QUALITY OF LIFE (TOTAL SCORE)			P-VALUE

		OPTIMAL (N = 50) FREQ (%)	SUB OPTIMAL (N = 29) FREQ (%)	POOR (N = 4) FREQ (%)	

Age, mean (SD), years	18.5(4.5)	18.2(4.7)	18.7(4.3)	19(2.7)	0.536

<19	42(50.6)	25(50)	14(48.3)	3(75)	0.600

>19	41(49.4)	25(50)	15(51.7)	1(25)	

Weight, mean (SD), kg	51.9(11.3)	49.4(11.3)	56.5(10.7)	50.8(7.1)	0.630

Height, mean (SD), cm	158.6(8.6)	158.3(8.8)	159.5(8.8)	156(7.1)	0.902

Sex					

Female	49(59)	26(52)	20(69)	3(75)	0.269

Male	34(41)	24(48)	9(31)	1(25)	

Level of education					

Pre–primary	4(4.8)	4(8)	0(0)	0(0)	0.344

Primary	36(43.4)	19(38)	090)	0(0)	

Secondary	39(47)	23(46)	14(48.3)	3(75)	

None	4(4.8)	4(8)	15(51.72)	1(25)	

Duration of heart disease from 1^st^ diagnosis, median (IQR) years	6(2–25)	11.5(8.8–15)	11(7–15.5)	14.5(11–18)	0.341

Duration from surgery, median (IQR), years	4(2–6)	4(2–6)	4(3–6.5)	3(1–5.8)	0.313

Time to surgery, median (IQR)	2(1–4.8)	2(1–4)	2(0.8–5)	2(0–3)	0.604

Body mass index (BMI), median (IQR)	20.1(12.7–31.6)	19(12.7–27.7)	20.7(16.9–31.6)	19.4(18.1–27.1)	**0.008**

Underweight (BMI<18.5)	26(31.3)	21(42)	4(13.8)	1(25(	**0.044**

Normal weight (18.5–24.9)	46(55.4)	25(50)	19(65.5)	2(50)	

Overweight (25.0–29.9)	9(10.8)	4(8)	4(13.8)	1(25)	

Obese (≥30.0)	2(2.4)	0(0)	2(6.9)	0(0)	

Country					

India	2(2.5)	1(2)	1(3.7)	0(0)	0.837

Uganda	6(7.5)	5(10.3)	1(3.7)	0(0)	

Sudan	71(88.8)	42(85.7)	25(92.6)	4(100)	

Valve replacement					

Single valve	58(28.4)	35(72.9)	20(69)	3(75)	0.922

Double valve	23(71.6)	13(27.1)	9(31)	1(25)	

Age of the surgery (IQR), years	14(7–18)	13.5(8–18)	7(3–20)	17(11–18)	0.120


**Table 4b:** Socio-demographic factors associated with optimal health-related quality of life of the participants at bivariate analysis.


VARIABLE	ALL (N = 83) FREQ (%)	HEALTH RELATED QUALITY OF LIFE (TOTAL SCORE)			P-VALUE

		OPTIMAL (N = 50) FREQ (%)	SUB OPTIMAL (N = 29) FREQ (%)	POOR (N = 4) FREQ (%)	

**Taking warfarin (n = 83)**					

I don’t mind taking warfarin					

Definitely false	38(45.8)	22(44)	15(51.7)	1(25)	0.556

Definitely true	45(54.2)	28(56)	14(48.3)	3(75)	

I can’t always be bothered taking my tabs					

Definitely false	62(74.7)	36(72)	22(75.9)	4(100)	0.457

Definitely true	21(25.3)	14(28)	7(24.1)	0(0)	

It interferes with my social life					

Definitely false	66(79.5)	41(82)	22(74.9)	3(75)	0.815

Definitely true	15(18.1)	8(16)	6(20.7)	1(25)	

Mostly false	1(1.2)	1(2)	0(0)	0(0)	

Mostly true	1(1.2)	0(0)	1(3.5)	0(0)	

I don’t fully understand why I have to take them					

Definitely false	70(87.5)	43(89.6)	23(82.1)	4(100)	0.774

Definitely true	4(5)	2(4.2)	2(7.1)	0(0)	

Mostly false	3(3.8)	1(2.1)	2(1.1)	0(0)	

Not sure	3(3.8)	2(4.2)	1(3.7)	0(0)	

I simply forget					

Definitely false	69(84.2)	45(91.8)	21(72.4)	3(75)	0.116

Definitely true	7(8.5)	2(4.1)	4(13.8)	1(25)	

Mostly false	3(3.7)	2(4.1)	1(3.5)	0(0)	

Mostly true	1(1.2)	0(0)	1(3.5)	0(0)	

Not sure	2(2.4)	0(0)	2(6.9)	0(0)	

**I am afraid I may get a bleed or bruise**					

Definitely false	53(64.6)	39(78)	13(46.4)	1(25)	**0.017**

Definitely true	24(29.3)	10(20)	11(39.3)	3(75)	

Mostly false	3(3.7)	1(2)	2(7.1)	0(0)	

Mostly true	1(1.2)	0(0)	1(3.6)	0(0)	

Not sure	1(1.2)	0(0)	1(3.6)	0(0)	

I don’t want my friends/colleagues to know I have an artificial valve					

Definitely false	47(56.6)	40(80)	7(24.2)	0(0)	**<0.001**

Definitely true	35(42.2)	10(20)	21(72.1)	4(100)	

Mostly true	1(1.2)	0(0)	1(3.5)	0(0)	

**Doing regular blood tests (n = 83)**					

I don’t mind having a regular blood test					

Definitely false	38(45.8)	22(44)	13(44.8)	3(75)	0.504

Definitely true	44(53.0)	28(56)	15(51.7)	1(25)	

Mostly false	1(1.2)	0(0)	1(3.5)	0(0)	

The tests are painful					

Definitely false	39(47)	26(52)	11(37.9)	2(50)	0.495

Definitely true	40(48.2)	20(40)	18(62.1)	2(50)	

Mostly false	2(2.4)	2(4)	0(0)	0(0)	

Not sure	2(2.4)	2(4)	0(0)	0(0)	

It interferes with my daily life					

Definitely false	63(75.9)	41(82)	20(69)	2(50)	0.303

Definitely true	17(20.5)	8(16)	7(24.1)	2(50)	

Mostly false	1(1.2)	0(0)	1(3.5)	0(0)	

Not sure	2(2.4)	1(2)	1(3.5)	0(0)	

I don’t understand why I have to have them					

Definitely false	72(86.8)	45(90)	23(79.3)	4(100)	0.322

Definitely true	8(9.6)	5(10)	3(10.3)	0(0)	

Mostly false	1(1.2)	0(0)	1(3.5)	0(0)	

Not sure	2(2.4)	0(0)	2(6.9)	0(0)	

It is expensive to me (I can’t afford it)					

Definitely false	38(46.3)	28(56)	8(28.6)	2(50)	0.089

Definitely true	41(50)	20(40)	19(67.9)	2(50)	

Mostly true	1(1.2)	0(0)	1(3.6)	0(0)	

Not sure	2(2.4)	2(4)	0(0)	0(0)	


At multivariable analysis, of the odds of having a better HRQoL increased by 20% for a very unit increase in BMI (aOR, 1.2; 95% CI: 1.1–1.3, p = 0.006). Those with normal BMI and overweight individuals had higher odds of having a better HRQoL; however, these increases were not statistically significant. Participants who were afraid of bleeding or bruising had 50% higher odds of having a better HRQoL (aOR: 1.5, 95% CI: 1.21–2.47, p = 0.004). Finally, participants who accept having an artificial valve in their body with had an almost 3-fold higher odds of having a better HRQoL compared to those who were hesitant (aOR: 2.7, 95% CI; 1.64–3.81, p < 0.001) (Table 5).

**Table 5:** Factors associated with optimal health-related quality of life of the participants at multivariable analysis.


VARIABLE	ODDS RATIO	95% CI	P-VALUE

BMI, median(range)	1.2	1.1–1.3	**0.006**

Underweight (<18.5)	1.0		

Normal weight (18.5–24.9)	1.3	0.68–2.66	**0.001**
	
Overweight (25.0–29.9)	1.6	0.162–2.42	**0.025**

Obese (>30.0)	N/A		

I am afraid I may get a bleed or bruise			

Definitely false	1.0		

Definitely true	1.5	1.21–2.47	**0.004**

Mostly false	1.5	-0.82–3.75	0.208

Mostly true	2.4	-1.54–6.36	0.231

Not sure	2.4	-1.54–6.36	0.231

I don’t want my friends/colleagues to know that i have an artificial valve			

Definitely false	1.0		

Definitely true	2.7	1.64–3.81	**<0.001**

Mostly true	3.3	-.0.83–7.5	0.116


## Discussion

In this study aimed at evaluating the HRQoL of children who have undergone VRS for complicated RHD in Uganda, HRQoL was optimal in about three in five participants following VRS. Furthermore, we found that increasing BMI was associated with a 20% improvement in HRQoL and acceptance of artificial valve improved HRQoL by almost threefold. This is the first study to assess the HRQoL of children in Uganda with RHD following VRS. However, whether the better HRQoL is due to VRS or not remains unknown, since no control groups were included in this study.

Our study revealed improvement in the HRQoL after VRS, the mean self-reported cardiac-specific HRQoL score was optimal 83.4 ± 12.9%. It was noted that not only symptoms and general wellbeing improved after valve replacement surgery, but also cognitive and communication problems improved with mean scores of 90.6 (±15.1), and 87.5 (±22.4) respectively. These findings were consistent with Parviz et al. [16] who reported valve surgery improved overall scores of HRQoL and was considered an effective strategy to improve the quality of life of patients with valve disease. In our study, the lowest score was the perceived physical appearance 55.8 (±41.8), similarly, a low score in this dimension was also reported [17]. This finding can be explained by the fact that the majority of our study participants were female and the presence of an open-heart surgery scar in the chest gave a negative perception of physical appearance, worse results in this domain were also found in Italian children and adolescent with congenital heart diseases [18]. When compared to HRQoL of children and adolescents with operated congenital heart disease in LMIC, our study participants demonstrated better scores in the heart problems, treatment anxiety and communication, similar in the treatment and cognitive problems but worse in perceived physical appearance [19]. We also found a positive correlation between the acceptance of the presence of an artificial valve in the body and optimal HRQoL (p > 0.001). All our participants had mechanical valves and they were subjected to daily warfarin use and regular monthly blood tests; however, we didn’t find a negative relationship toward the HRQoL. Participants who were afraid of getting bleeding or bruising from warfarin use had better HRQoL (p = 0.004). Most of our participants were either in primary or secondary schools and the mean age of the participants at the time of the study was 18.5 years (SD 4.5), implying a delay in the level of education compared to the average age (18 years) of the entrance to university in Uganda.

In general, regular blood testing was not well tolerated, a significant number of participants reported the tests were causing pain, and about half of the participants reported these being expensive (this cost was not covered in the Salam center project). This may be improved by making ‘free self-test kits’ [20,21], available and introducing a self-management system for monitoring oral anti-coagulation and it might reduce the need for patients to go to the hospital for blood sampling and dose adjustment, saves time and effort and requires less resources especially in LMIC where the laboratories are seldom available, despite studies demonstrated the feasibility of self-management and monitoring of oral anti-coagulation therapy [22,23,24,25], it is not yet in widespread use.

Usually, the success of surgical interventions and treatments has been measured based on mortality and morbidity outcomes from the physician’s perspective [26]; however, assessing the HRQoL using available validated researcher/self-administered questionnaires from the patients perspective may help in identifying hidden morbidities or challenges that might be addressed by the healthcare provider, assist in clinical decision-making [11] and may give better patient-physician communication.

This study has important limitations. Firstly, this was a single-center study at a national center of excellence in cardiac care. Therefore, findings may not be generalizable to patients receiving care from lower facilities. However, the RHD registry has data from across the country and we contacted children on the registry to provide more robust and generalizable results. The study was conducted during COVID-19 pandemic where the COVID stringency index for Uganda ranged from 37.96 to 87.04 [27,28], that could also potentially impact on the HRQoL since stringency of governmental measures against the spread of COVID-19 varied over time. Secondly, we did not include a control population of either patients with unrepaired RHD or children without heart disease. Therefore, our findings may not reflect the true impact of RVS to the HRQoL of these patients. However, this study provides baseline findings for future larger studies. Thirdly, we used a cross-sectional design, and yet HRQoL of life may change over time. Additionally, data was not supported by blood tests or imaging and other treatments that might have a significant impact on the quality of life. Fourthly, PEDSQoL has not been validated in Uganda for the study of patients with RHD and following VRS. However, other studies abroad have validated this tool for the same diagnosis and age group.

## Conclusion

In this study at a national heart treatment center in Uganda, HRQoL was optimal in about three in five participants following VRS for complicated RHD. Most patients had these surgeries performed outside Uganda. Increasing BMI and acceptance of artificial valve were significantly associated with optimal HRQoL. Optimization of nutrition is key to improve HRQoL in this population, in addition, psycho-social support for enhanced adherence to long-term warfarin therapy and INR monitoring as well as acceptance of artificial valves may further improve HRQoL in this population.

## Data Accessibility Statements

All data are available upon reasonable request from the first author.
